# Effects of Music Aerobic Exercise on Depression and Brain-Derived Neurotrophic Factor Levels in Community Dwelling Women

**DOI:** 10.1155/2015/135893

**Published:** 2015-05-14

**Authors:** Shu-Hui Yeh, Li-Wei Lin, Yu Kuan Chuang, Cheng-Ling Liu, Lu-Jen Tsai, Feng-Shiou Tsuei, Ming-Tsung Lee, Chiu-Yueh Hsiao, Kuender D. Yang

**Affiliations:** ^1^Department of Medical Research and Development, Show Chwan Memorial Hospital, Changhua 500, Taiwan; ^2^Department of Nursing, Central Taiwan University of Science & Technology, Taichung 400, Taiwan; ^3^Department of Nursing, Hungkuang University, Taichung 433, Taiwan; ^4^Yin Huo Health Association, Kaohsiung 806, Taiwan; ^5^School of Nursing, National Taichung University of Science & Technology, Taichung 403, Taiwan; ^6^Department of Nursing, Tzu Hui Institute of Technology, Pingtung County 926, Taiwan; ^7^Institute of Clinical Medicine, National Yang Ming University, Taipei 112, Taiwan

## Abstract

A randomized clinical trial was utilized to compare the improvement of depression and brain-derived neurotrophic factor (BDNF) levels between community women with and without music aerobic exercise (MAE) for 12 weeks. The MAE group involved 47 eligible participants, whereas the comparison group had 59 participants. No significant differences were recorded in the demographic characteristics between the participants in the MAE group and the comparison group. Forty-one participants in the MAE group and 26 in the comparison group completed a pre- and posttest. The MAE group displayed significant improvement in depression scores (*p* = 0.016), decreased depression symptoms in crying (*p* = 0.03), appetite (*p* = 0.006), and fatigue (*p* = 0.011). The BDNF levels of the participants significantly increased after the 12-week MAE (*p* = 0.042). The parallel comparison group revealed no significant changes in depression scores or BDNF levels. In summary, the 12-week MAE had a significant impact on the enhancement of BDNF levels and improvement of depression symptoms. Middle-aged community women are encouraged to exercise moderately to improve their depression symptoms and BDNF levels.

## 1. Introduction

Depression is prevalent in community residents and affects about 121 million people worldwide. Fewer than 25% of those affected have access to effective treatments [1]. Our previous research reveals that 27.5% of community dwelling elders in Taiwan have depression symptoms [2]. The prevalence of depression symptoms in community dwelling women is increasing [3]. The lifetime risk of depression in people who are healthy enough to survive to old age is 23% in men and 45% in women [4]. Aging, being female, having a low social economic status, joblessness, separation or divorce, and having a family history of depression are among the risk factors for depression [5].

Middle-aged and aged women with depression who experience depressed moods, loss of interest or pleasure, feelings of guilt or low self-worth, disturbed sleep or appetite, low energy, and poor attention have lower quality of life [6, 7]. These depression symptoms can become chronic or recurrent and lead to substantial physical inactivity and poor family functions [8]. The caregiving role of women for family members, such as children and elders, can restrict time for exercise. Cultural expectations can also limit the participation of community dwelling women in certain physical activities. Physical inactivity in women can also affect their personal relationship and life quality [9]. Therefore, physical inactivity is generally more prevalent among women than men [10]. Women usually report spending less time participating in leisure activities than men in the Organization for Economic Cooperation and Development (OECD) countries [11]. Women in these countries spent 22 to 80 minutes less in leisure time a day than men. Physical inactivity has been shown to correlate inversely to mental functions [12]. Aged women with depression have a lower quality of life and may develop chronic symptoms of physical inactivity. Therefore, it is essential to develop physical activities, with a leisure component such as music, for community dwelling women.

*Physical Activity, Depression, and BDNF*. Aerobic exercise is known to enhance mood and cognitive function in humans, although the physiological mechanisms of these effects remain unclear [13]. Our previous study showed that regular tai chi chuan exercise for 12 weeks promoted functional mobility and self-health perception [14]. It is postulated that appropriate exercise may be an adjunct treatment to relieving symptoms of depression. Some studies have shown positive effects of physical exercise on improvement of depression [15] and cognitive performance [16]. Music intervention, listening to or playing a musical instrument, has also been shown to improve depression and cognitive function [17, 18]. Gerra et al. [19] found that different types of music have varied effects on the auditory and endocrine systems. Music melody and rhythm can stimulate nerve impulses through the limbic system, the thalamus, and reticular activating system (RAS), thereby affecting the neuroendocrine system in humans [20]. Recently, exercise has been shown to increase brain-derived neurotrophic factor (BDNF) in aged women [21, 22]; moreover, exercise has been also shown to increase BDNF levels in aged women with recurring major depression [23]. Recently, Satoh et al. [24] reported that physical exercise with music improved cognitive function of elderly people.

Taken together, we propose a research framework that community dwelling women may respond to an exercise program with rhythmic music that stimulates increased BDNF and immune parameters resulting in a reduction in depression symptoms. We developed a moderate exercise program that integrates rhythmic music, called music aerobic exercise (MAE), with a maximal heart rate (HRmax) of 64% and stimulation of certain immune parameters in middle-aged women [25]. This study was further broadened to investigate whether MAE could improve depression symptoms and blood BDNF levels in community dwelling women after a 12-week MAE program.

## 2. Methods

### 2.1. Study Design

A cluster random sampling method was used to recruit participants from central and southern Taiwan. The sample size was initially estimated to reach a power of 0.8, one-tailed *α*-level of 0.05, effect size of 0.36, and 45 participants for each group, with an additional 18% to account for withdrawal. In total, 106 participants from four communities were recruited (communities were located within a one-hour car ride from where the MAEs were held).

### 2.2. Subjects and Data Collection

**Figure 1:**
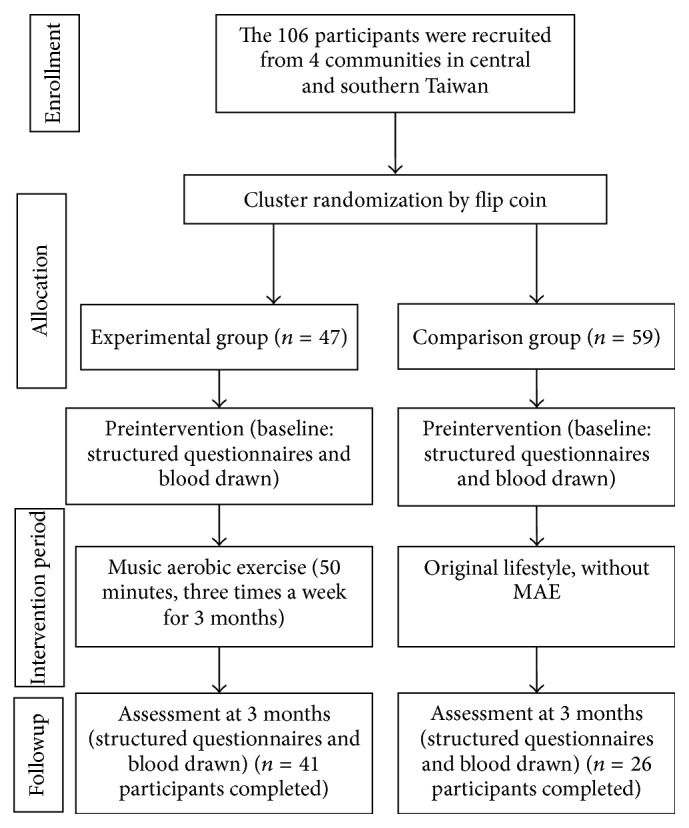
Flow chart depicting the trial.

Participants were recruited according to the following inclusion criteria: (1) living in central and southern Taiwan, (2) residing in one of the selected communities, (3) being 40 years old or older, (4) being able to communicate in Mandarin or Taiwanese, and (5) willing to sign an informed consent before participating in this study. Exclusion criteria of participants included (1) severe cognitive deficits screened by using the short portable mental status questionnaire (errors of SPMSQ ≥4), (2) major cardiovascular disease screened prior to the exercise, and (3) use of antidepressants. Participants were assigned randomly into experimental and comparison groups, with and without exercise three times a week, after informed consent was signed. The participants were also informed that the MAE exercise was specifically for the experimental group and no specific exercise for the control group was required during the study course. Pre- and postexercise tests included depression scores and BDNF levels. Modifications for standardization of the measures and measurement protocol were derived from our previous publication [25]. After the institutional review board approved the study proposal, middle-aged and aged women were recruited from four separate communities to participate in this study. By the end of the study, the withdrawal rate was 36.8% (Figure 1) possibly because the participants in the comparison group did not enjoy watching TV and would have rather been in the exercise group.

To reach a power of 0.8, effect size of 0.36, and 45 participants for each group, with an additional 18% to account for withdrawal, we recalculated the one-tailed alpha level as 0.045 and set the null hypothesis of significant alpha level at <0.045.

Participants received blood draws (3 mL) and were screened for depression at the beginning and end of the 12-week MAE program to detect changes in the outcome indicators. A clinical study nurse conducted a structured questionnaire interview (25 min) and blood draw (10 min) in each participant's community. This process took about 35 minutes for each participant. The experimental group received a regular schedule of MAE (50 minutes, three times each week for three months), whereas the comparison group followed their regular activities of daily living and watched 50 minutes of television three times each week. In the pretest, the study nurses and medical technologists who carried out the assessments were blinded. But, in the posttest, only the medical technologists who carried out the blood analysis were blinded.

### 2.3. Research Instruments

Three structured questionnaires including SPMSQ [26], demographic questionnaire, and Beck Depression Inventory II (BDI-II) [27] were used for data collection. The SPMSQ was used to screen the cognitive function of the participants. The measurement tools are described as follows.

#### 2.3.1. Short Portable Mental Status Questionnaire

The SPMSQ was originally developed by Pfeiffer [26] and has been widely used in different studies as a cognitive screening tool. The SPMSQ was used to screen the participants' cognitive function before the interview for inclusion criteria and randomization. We set the criteria of the errors of SPMSQ <4 for eligibility and all participants studied had the errors of SPMSQ ≤3.

#### 2.3.2. Demographic Questionnaire

The demographic questionnaire was used to record information on age, gender, educational level, marital status, number of children, number of chronic diseases, body weight, height, number of years they have lived in their community, and perceived income adequacy (1 = extremely inadequate and 5 = extremely adequate with ability to save money).

#### 2.3.3. Beck Depression Inventory II

The original BDI was developed by Beck et al. [28]. The BDI-II was modified by Beck et al. in 1996 and is a widely used rating questionnaire for depression resulting in a global score between 0 and 63. The BDI-II consists of 21 questions and has been translated into different languages, including Chinese. The questionnaire is easy to handle and can be completed within five to ten minutes [29] and has been used for a wide range of the participants in different settings with good reliability and validity [27, 30, 31]. In the Chinese version, the study was conducted with 180 outpatients with Cronbach's *α* level = 0.94 and split-half reliability = 0.91 [32]. BDI-II has been used in pre- and postpartum depression screening among Chinese women and demonstrated that the results could be divided into severe and mild depression. The cut point score was 10, selecting participants with severe depression with a sensitivity of 100% and specificity of 78.6%, mild depression with a sensitivity of 83%, and specificity of 86%, respectively [33]. To exclude potential confounders who have severe depression, we set the exclusion criteria of BDI score >13. Finally, we found that all the participants studied had the BDI score <13.

#### 2.3.4. Luminex Multiplex Assay of Peptide Hormone (BDNF)

The BDNF levels were measured through Luminex^100^ assay. A standard capture sandwich assay was developed with the Luminex Flow Metrix system (Luminex, Austin, TX, USA) to determine the different levels of BDNF concentrations in serum samples. The anti-BDNF antibody was coupled to a different bead set (#HBDP-33K, Milliplex MAP, USA) for this assay. The assay was performed in our immunology laboratory where we routinely measure immune parameters and other serum factors using 50 *μ*L for the measurement [34]. The accuracy and precision of the Luminex 100 measurement were reported as having a misclassification of microspheres <0.5%.

### 2.4. Contents and Practice of MAE

A regular schedule of MAE (50 minutes, three times each week for three months) was used for the intervention. The MAE was developed by one of the coinvestigators six years ago and was referred to as “rhythm movement through music temperament” that incorporates music rhythm and postural movement into exercise [25]. Eleven items of music with exercise, including stretching exercise, flexibility exercise, body circulation exercise, skillful hand and active brain exercise, stepping exercise with music, aerobic exercise with music, slow walking exercise, relaxed turning exercise, hand butterfly (enhancing hand muscle strength), meditation, and breathing exercise, were included in the MAE. Each 50-minute MAE session included a warm-up stage with two quiet music songs, an active exercise with six faster rhythms, and a cool-down stage with two quiet music songs [25]. Five experts in sports science and five experts in music exercise validated and standardized the MAE protocol. Instructions on how to prevent sports injuries were given to the participants prior to the program. The research staff and instructors were trained to lead MAE before the intervention began.

The exercise intensity of the MAE session was geared towards achieving moderate exercise with an HR_max⁡_ of 64% calculated by using peak HR ÷ (220 − age) × 100% [35]. In this exercise program, the resting heart rates (before MAE exercise) and maximal heart rates during the warm-up and active aerobic segment were measured in 10 community dwelling women through the use of wireless pulse oximeters with heart rate monitors.

### 2.5. Research Procedures

One research associate and two assistants were trained for four weeks before data collection. Interrater reliability was 0.91. Ethical considerations were addressed during the study. Women from the four communities were approached individually by the well-trained research associate and assistants. The investigator supervised the research assistants during the study and data collection process. After determining whether the women met the inclusion criteria, the research assistant described the study in Mandarin and Taiwanese and obtained informed consent before the study.

### 2.6. Ethical Considerations

Prior to the study, the institutional review board approved the research proposal. All participants were informed of their rights and were asked to sign a consent form prior to participating in this study. Participants were assured anonymity. The women participating in this study were informed that they could withdraw from the study at any time.

### 2.7. Statistical Analysis

The data were coded, double-checked, and analyzed using the Statistical Package for Social Sciences for Windows Version 22.0 (SPSS Inc., Chicago, IL, 2013). In addition to descriptive statistics, baseline measures between the two groups were compared through* t*-tests, the Chi-square test, and Fisher's exact test, where appropriate. Changes in the various outcome measures between two groups over the duration of the study period were analyzed by using a mixed-design analysis of variance model.

## 3. Results

### 3.1. Flow Chart of Completion and Drop-Out of the Participants

A cluster randomized control trial design was utilized. One hundred and six participants from four communities were assigned to either an experimental group with MAE or a comparison group (50 minutes of TV watching), as shown in Figure 1. Forty-seven eligible participants were enrolled into the experimental group with a weekly MAE schedule, and 59 participants without MAE were enrolled in the comparison group. Among the 47 participants in the experimental group, 41 completed the program and pre- and posttest (depression questionnaire and blood BDNF levels). Participants in the comparison group only attended to watch TV for 50 minutes without exercise three times a week. Only 26 of the 59 participants in the comparison group completed the entire 12-week program and posttest possibly because this group did not enjoy watching TV and would have rather been in the exercise group. The demographic data between the participants who completed the program and those who did not revealed no differences in marital status, educational level, depression status, smoking, alcohol use, chronic illness, unemployment, menopause, perceived health, height, or weight (Table 1). The demographic data between experimental and comparison groups revealed insignificant differences on all items (Table 2).

### 3.2. Changes of Overall Depression Symptoms

The overall depression scores in the experimental and comparison groups were 5.18 ± 5.91 (range: 2–12) and 4.52 ± 4.12 (range: 2–11), respectively. These scores fell into the no depression to mild depression category, as defined by the study by Pfeiffer [26].  Table 3 shows that participants in the MAE displayed significant improvements in depression scores (*p* = 0.016) in contrast to the comparison group that demonstrated no significant difference in overall depression scores.

### 3.3. Changes of Individual Depression Symptoms

The Chinese version of the modified BDI was used to assess depression symptoms and included 21 items. Interestingly, we found the scores of 19 of the 21 items decreased after the MAE program, although only 3 items reached a level of significance (Table 4). The significant reduction in symptom scores in the MAE group included crying (*p* = 0.03), changes in appetite (*p* = 0.006), and fatigue (*p* = 0.011). By contrast, depression symptom scores in the comparison group revealed no significant differences.

### 3.4. Changes of BDNF Levels Pre- and Postexercise

**Figure 2:**
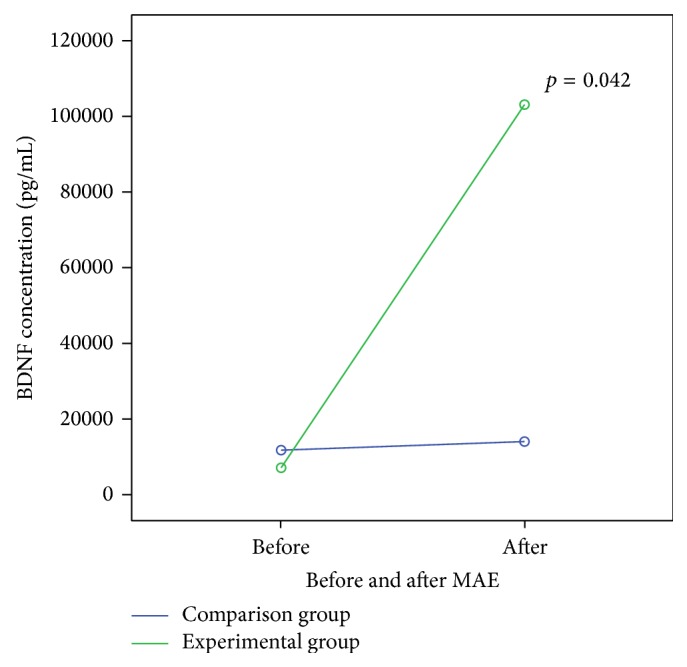
The changes in BDNF levels before (pretest) and after (posttest) MAE between experimental and comparison groups. Data were analyzed by using mixed-design analysis of variance model.

The study results showed that BDNF levels were not significantly different between the experimental and comparison groups in the pretest. The BDNF levels, however, significantly (*p* = 0.002) increased after the 12-week MAE in the middle-aged and aged women (Figure 2). By contrast, the BDNF levels in the comparison group revealed insignificant changes from the pretest to posttest. The data derived from the community dwelling women suggested that moderate exercise, such as a 12-week MAE, could improve depression symptoms, presumably through an increase in BDNF levels.

## 4. Discussion

We developed a moderate exercise program with music called music aerobic exercise (MAE), which is easy to follow and provides exercise for community dwelling women. Employing the MAE, we reported that the MAE with an average HR_max⁡_ of 64% induced Foxp3 transcription factor expression compatible to increase CD4CD25 lymphocytes [25]. In this study, we further demonstrated that the MAE ameliorated depression symptoms, especially in reducing crying, changes in appetite, and fatigue, and increased blood levels of BDNF. We were the first to discover that rhythmic moderate exercise promoted T cell regulatory function, improved depression symptoms, and increased levels of BDNF.

Different types of exercise have a wide variety of effects on immunity. Exhaustive exercise has been shown to cause inflammatory reaction and immunosuppression associated with risk of upper respiratory tract infections [36]. In contrast, certain moderate exercises benefit physical fitness and immunity [37]. Improved mood enhances immunity and reduces allergic disease [38]. Thus, we combined music with moderate aerobic exercise to benefit human immunity and mental functioning. Music can influence the physiological and psychological responses of its listeners. Some kinds of music can even relieve pain, reduce anxiety, and promote sleep quality. In the experimental group, subjects who listened to their choice of five different 30-minute music pieces in the morning for two consecutive weeks had improved depression scores [20]. We found that the combination of music and exercise enhanced immunity, induced the release of beneficial hormones such as BDNF, and decreased depression symptoms.

BDNF has been demonstrated to play a critical role in the activity-dependent processes, including synapse development and plasticity [39]. A previous study has reported that BDNF, by acting via the protein tyrosine kinase receptor [40], regulated a number of processes including neuronal development and its functions. BDNF is also involved in memory formation, including learning and behavior, synaptic plasticity, synaptic efficacy, and neuronal connectivity. BDNF levels in patients suffering from major depression disorders are significantly lower than control subjects [41]. Low plasma BDNF has also been reported to be associated with suicidal behavior in depressed patients [42]. Interestingly, patients with depression who received an 8–12-week course of treatment with antidepressant medications exhibited a significant increase in the serum BDNF concentration [41]. Basal BDNF in patients suffering from posttraumatic stress disorder was significantly lower than in healthy individuals [43]. Exercise is one of the proposed alternative therapies to promote physical and mental health for individuals with depression symptoms. However, most studies examining the effects of exercise on mental health have been conducted with a small sample size of college students in school settings or adults in hospital settings. Few studies have been conducted in randomized trials intended to measure physiological indices. A recent study that followed 3500 elders (>64 years of age) for eight years in the United Kingdom showed that exercise, regardless of what age it began, significantly improved physical function [44]. Researchers have a growing interest in studies concerning the effect of physical exercise and training on the functioning of the brain, with special focus on its effect on BDNF [45]. They found that exercise could increase the neurotransmission of a neural substance, BDNF, which has been proven to reduce the effects of depression and anxiety as well as bringing comfort. Several studies indicated that exercise induced an upregulation of the BDNF in the hippocampus and thus could play an important role in the enhancement of cognitive function in humans [45]. Our research results are consistent with the above studies.

## 5. Conclusions

The results of this study showed that MAE improved depression symptoms and increased neurorelated hormones, such as BDNF in community dwelling women. During the study period, we kept a diary to record possible situations or events that could affect the study results and potentially contribute to possible side effects. No Hawthorne effect, historical events, exercise injury, or mortality was reported during the research period. This study model can be developed into a standardized protocol and utilized to promote mental and physical health for community dwelling women. Administrators, clinical practitioners, and researchers can build cooperative teams to improve the mental and physical health of women. Based on these study results, we will establish an MAE customized for wheel-chair dependent elders to promote better physical, psychological, and immunological health for women living in long-term care facilities.

The study was limited to community dwelling women. Therefore, the results from this study cannot be generalized to the general population. Although we kept a diary to record possible situations and side effects during the study course, we did not collect nonstudy related exercise activities of the study and comparison participants during the intervention period. Data on physical activity levels prior to this study were not collected in this study. Moreover, the comparison group had a lower completion rate at 26/59 because they did not enjoy the 50-minute TV sessions, 3 times a week for 12 weeks. Since watching TV is not a comparative activity, further studies should define a better design to simultaneously compare the effects of exercise, music, and their combination on the improvement of depression and changes of BDNF levels.

## Figures and Tables

**Table 1:** Comparisons of demographic characteristics between drop-out and completed participants.

	Drop-out participants			Completed participants				
Variables	*N* = 39			*N* = 67			*χ* ^2^ ^a^/*t* ^b^	*p*
	*n*	%	M ± SD	*n*	%	M ± SD		
Age			53.4 ± 9.9			52.7 ± 10.9	0.31^b^	0.76
Marital status							0.95^a^	0.69
Unmarried	2	5.1		7	10.4			
Married	27	69.2		45	67.2			
Others	10	25.6		15	22.4			
Educational level							5.15^a^	0.08
Elementary	8	20.5		21	31.8			
High school	26	66.7		29	43.9			
College, above	5	12.8		16	24.2			
Diagnosed depression							0.02^a^	1.00
None	38	97.4		65	97.0			
Yes	1	2.6		2	3.0			
Smoking							4.21^a^	0.06
None	35	89.7		66	98.5			
Yes	4	10.3		1	1.5			
Alcohol use							1.41^a^	0.27
None	31	79.5		59	88.1			
Yes	8	20.5		8	11.9			
Chronic illness							0.99^a^	0.42
None	19	48.7		26	38.8			
Yes	20	51.3		41	61.2			
Employed status							0.05^a^	0.84
None	23	59.0		38	56.7			
Yes	16	41.0		29	43.3			
Menopause							0.03^a^	1.00
None	20	51.3		35	53.0			
Yes	19	48.7		31	47.0			
Hormone therapy							1.34^a^	0.32
None	37	94.9		59	88.1			
Yes	2	5.1		8	11.9			
Exercise per week (mins)			74.6 ± 138.1			58.4 ± 105.5	0.67^b^	0.51
Perceived health			69.6 ± 14.4			62.8 ± 18.8	1.94^b^	0.06
Height (cm)			156.4 ± 5.9			156.8 ± 6.3	−0.30^b^	0.76
Weight (Kg)			61.1 ± 7.5			61.4 ± 11.9	−0.13^b^	0.90

*χ* ^2^ ^a^: chi-square values analyzed by using chi-square test. Fisher's exact test was used when the expected value was less than 5. *t* ^b^: *t* values analyzed by using *t*-test.

**Table 2:** Comparisons of demographic characteristics between comparison and MAE groups.

	Comparison group			MAE group				
Variables	(*N* = 26)			(*N* = 41)			*χ* ^2^ ^a^/*t* ^b^	*p* value
	*n*	%	M ± SD	*n*	%	M ± SD		
Age	26		51.9 ± 11.9	41		53.2 ± 10.3	−0.51^b^	0.61
Marital status							1.20^a^	0.59
Unmarried	4	15.4		3	7.3			
Married	17	65.4		28	68.3			
Others	5	19.2		10	24.4			
Educational level							1.77^a^	0.40
Elementary	6	24.0		15	36.5			
High school	11	44.0		18	43.9			
College, above	8	32.0		8	19.5			
Diagnosed depression							1.31^a^	0.52
None	26	100.0		39	95.1			
Yes	0	0		2	4.9			
Smoking							1.60^a^	0.39
None	25	96.2		41	100.0			
Yes	1	3.8		0	0			
Alcohol use							0.01^a^	1.0
None	23	88.5		36	87.8			
Yes	3	11.5		5	12.2			
Chronic illness							0.06^a^	1.0
None	10	38.5		17	41.5			
Yes	16	61.5		24	58.5			
Employed status							0.78^a^	0.44
None	18	69.2		24	58.5			
Yes	8	30.8		17	41.5			
Menopause							0.16^a^	0.80
None	13	50.0		22	55.0			
Yes	13	50.0		18	45.0			
Hormone therapy							0.73^a^	0.47
None	24	92.3		35	85.4			
Yes	2	7.7		6	14.6			
Perceived health			65.2 ± 16.1			61.4 ± 20.3	0.79^b^	0.43
Height (cm)			155.7 ± 6.0			157.4 ± 6.5	−1.07^b^	0.29
Weight (Kg)			58.8 ± 10.4			63.1 ± 12.6	−1.44^b^	0.16

*χ* ^2^ ^a^: chi-square values analyzed by using chi-square test. Fisher's exact test was used when the expected value was less than 5. *t* ^b^: *t* values analyzed by using *t*-test.

**Table 3:** Comparisons of depression scores between comparison group and MAE group.

	MAE group		Comparison group			
Variables	(*N* = 41)		(*N* = 26)		*F* ^a^	*p* value
	Mean	SD	Mean	SD		
Total depression scores					6.15	0.016
Pretest	5.18	5.91	4.52	4.12		
Posttest	3.39	3.64	5.92	6.83		

^*a*^ *F* value analyzed by mixed-design analysis of variance model.

**Table 4:** Pre- and posttest depression scores in MAE and comparison groups.

	MAE group (*N* = 41)				Comparison group (*N* = 26)					
Variables	Pretest		Posttest		Pretest		Posttest		*F* ^a^	*p*
	Mean	SD	Mean	SD	Mean	SD	Mean	SD		
Sadness	0.17	0.495	0.02	0.156	0.08	0.272	0.12	0.326	2.51	0.12
Pessimism	0.17	0.381	0.05	0.218	0.23	0.514	0.27	0.533	1.99	0.16
Past failure	0.17	0.495	0.10	0.374	0.08	0.272	0.23	0.652	3.30	0.07
Loss of pleasure	0.17	0.495	0.15	0.422	0.15	0.368	0.27	0.533	0.73	0.396
Guilty feelings	0.20	0.401	0.10	0.300	0.12	0.326	0.08	0.272	0.25	0.62
Punishment feelings	0.10	0.300	0.10	0.300	0.04	0.200	0.12	0.326	0.65	0.42
Self-dislike	0.17	0.587	0.05	0.218	0.08	0.277	0.23	0.652	3.63	0.06
Self-criticalness	0.17	0.442	0.12	0.331	0.12	0.332	0.15	0.464	0.61	0.44
Suicide thoughts	0.13	0.335	0.05	0.218	0.12	0.440	0.08	0.272	0.10	0.75
Crying	0.29	0.716	0.07	0.264	0.08	0.272	0.23	0.710	4.74	0.03
Agitation	0.15	0.422	0.12	0.331	0.27	0.452	0.35	0.846	0.56	0.46
Loss of interest	0.17	0.442	0.15	0.358	0.15	0.368	0.19	0.402	0.23	0.64
Indecisiveness	0.22	0.475	0.10	0.300	0.08	0.272	0.12	0.326	1.99	0.16
Worthlessness	0.27	0.633	0.10	0.300	0.12	0.326	0.23	0.710	3.46	0.07
Loss of energy	0.46	0.674	0.34	0.480	0.38	0.496	0.50	0.583	1.88	0.18
Changes in sleeping pattern	0.44	0.673	0.37	0.536	0.38	0.637	0.38	0.752	0.12	0.73
Irritability	0.22	0.475	0.12	0.331	0.19	0.491	0.19	0.491	0.85	0.36
Changes in appetite	0.37	0.581	0.07	0.264	0.27	0.452	0.38	0.571	8.15	0.006
Concentration difficulty	0.17	0.381	0.17	0.381	0.31	0.471	0.31	0.471	0.00	1.00
Fatigue	0.37	0.488	0.27	0.449	0.38	0.496	0.64	0.638	6.86	0.011
Loss of interest in sex	0.95	0.944	0.78	1.061	0.92	1.060	0.96	1.172	0.28	0.60

^a^Groups compared with mixed-design analysis of variance model.
